# Social Networks Use Disorder and Associations With Depression and Anxiety Symptoms: A Systematic Review of Recent Research in China

**DOI:** 10.3389/fpsyg.2020.00211

**Published:** 2020-02-21

**Authors:** Zaheer Hussain, Elisa Wegmann, Haibo Yang, Christian Montag

**Affiliations:** ^1^School of Social Sciences, Nottingham Trent University, Nottingham, United Kingdom; ^2^General Psychology: Cognition and Center for Behavioral Addiction Research, University of Duisburg-Essen, Duisburg, Germany; ^3^Academy of Psychology and Behavior, Tianjin Normal University, Tianjin, China; ^4^Molecular Psychology, Institute of Psychology and Education, Ulm University, Ulm, Germany; ^5^neuSCAN Laboratory, MOE Key Laboratory for Neuroinformation, The Clinical Hospital of Chengdu Brain Science Institute, University of Electronic Science and Technology of China, Chengdu, China

**Keywords:** addiction, WeChat®, Weibo®, Social Networking Sites, depression, anxiety

## Abstract

**Background:** An increasing number of studies have investigated Social Networks Use Disorder (SNUD) among Western samples. In this context, the investigation of SNUD in Asia and especially in China has been much neglected. This poses a gap in the literature; it has been estimated that more than one billion Chinese people are using Chinese social networking sites (SNSs). Of note, many of these Chinese SNSs are rather unknown to researchers in Western countries.

**Aims:** The primary objective of the present systematic review was to identify and evaluate studies that investigated Chinese SNS use and associations between SNUD and depression and anxiety symptoms.

**Method:** A comprehensive search strategy identified relevant studies in PsycINFO, PsycARTICLES, Psychology and Behavioral Sciences Collection, MEDLINE, ProQuest, Web of Science, PubMed, Google Scholar, and the Chinese National Knowledge Infrastructure database (CNKI).

**Results:** The search strategy identified 35 potential studies, 13 studies were identified after shortlisting and full-text reviews of the studies, and finally 10 studies were included in the full review. Associations between SNUD, depression, and anxiety were reported in 10 studies. In eight (of the 10) studies, symptom severity of SNUD was associated with depression. Four studies reported associations between SNUD and anxiety. Most studies had utilized cross-sectional survey designs.

**Conclusions:** Most associations were found between SNUD and depression symptoms, but effect sizes were higher between SNUD and anxiety symptoms. The results have the potential to inform prevention and interventions on SNUD in Eastern cultures, although we explicitly state that our work focuses on China, the transfer of the present observations to other Asian countries (and their cultures) still needs to be established.

## Introduction

In 2012, China already had one of the world's most active environments for social networking site (SNS) use with more than 300 million users (Chiu et al., 2012). In 2014, there were more than 480 million Chinese SNS users (Socialmediatoday.com, 2014). By December 2018, the number of Internet users in China had reached 829 million, with a penetration rate of 59.6%, and the proportion of Internet users using smartphones is currently 98.6% (China Internet Network Information Center, 2019). Mak et al. (2014) reported that 70% of participants from the Chinese mainland and 65% from Hong Kong use SNSs suggesting that the use of SNSs is increasing rapidly in China. There are different SNSs used in China, among these are WeChat®, Weibo®, Qzone®, and QQ® (see also recent developments with the TikTok® platform showing huge growth rate in user numbers). The use of WeChat® has gained substantial popularity in China due to its multi-purpose character including payment functions and combining features of WhatsApp® and Facebook® (Gao and Zhang, 2013: Lien and Cao, 2014; Sampasa-Kanyinga and Hamilton, 2015; Montag et al., 2019a). According to statista.com, it has been reported that there are over one billion active WeChat® users (Statista.com, 2019) and it had reached 1.082 billion users by January 2019 (Weixin.qq.com., 2019), thereby showing the dramatic increase from the 300 million users in 2012. Weibo® represents a micro-blog platform and allows users to make 140-character posts, similar to Twitter®, it focuses on the sharing of opinions and information exchange (Sullivan, 2013). Qzone® is built around basic information presented by users, as well as pictures, comments, and videos posted by users and their friends (Apaolaza et al., 2014). QQ® consists of a large virtual community merged with interactive channels of searching, blogging, gaming, transactions, and social networking (Huang et al., 2013).

Given that, researchers should abstain from overpathologizing everyday life behaviors (Billieux et al., 2015), it is important to mention that SNS use can have positive effects on well-being. In this context, research observed that non-problematic use of SNSs was associated with higher external locus of control, greater online social interaction skills and higher life satisfaction (Nadkarni and Hofmann, 2012; Liu et al., 2016; Hou et al., 2017; Zhou et al., 2017). Active (vs. passive) use of SNSs together with meaningful interaction between people represents non-problematic use of SNSs (e.g., Escobar-Viera et al., 2018). In this context, research (Allen et al., 2014; Siddiqui and Singh, 2016) has reported that SNSs can improve social connectedness among users and can facilitate in the sharing of ideas between people and businesses across geographical boundaries. In this context, users do not experience negative consequences due to the usage of those platforms but rather experience the achievement and gratification of specific goals and needs. The term “problematic” is in itself problematic, because it is not clear if it describes the end of the spectrum or a transit zone from healthy via problematic to psychopathological SNS use. In any case, passive SNS use could cause mental health problems, in particular when users engage in upward social comparison processes (Tiggemann and Polivy, 2010; Vogel et al., 2014, 2015; Appel et al., 2015). Moreover, there are a growing number of individuals suffering from negative consequences due to the use of SNSs and given these potential negative outcomes, studies have shown associations between problematic SNS use and a range of mental health problems (Sampasa-Kanyinga and Hamilton, 2015; Sun et al., 2016). Recent research proposes that problematic SNS use or excessive overuse might even represent a distinct potential mental health problem (e.g., Balci and Gölcü, 2013; Montag et al., 2017, 2018b; Marino et al., 2018; Sha et al., 2019). Therefore, Van Rooij et al. (2017) called for the examination of specific online behaviors, such as problematic SNS use that show similarities to other addictive Internet-use patterns [see also exemplarily works by Montag and Becker (2018), Potenza et al. (2018), Sariyska et al. (2015), Tang et al. (2017), Tateno et al. (2018), Montag et al. (2015), Müller et al. (2017) and Wegmann et al. (2018)]. Andreassen and Pallesen (2014) describe the excessive, uncontrolled, or problematic use of SNS as “being overly concerned about SNSs, driven by a strong motivation to log on to or use SNSs, and to devote so much time and effort to SNSs that it impairs other social activities, studies/job, interpersonal relationships, and/or psychological health and well-being” (p. 4054). However, despite the initial state of research, to date there has been no classification of the disorder or specific terminology for it. Past research has used various terms such as Facebook addiction, social media addiction, problematic social-networks use, Internet-communication disorder, and social networks use disorder (SNUD; Montag et al., 2019b). We prefer the term SNUD, which is based on the terminology and the definition of gaming disorder in the ICD-11 of the World Health Organization (Pontes et al., 2019; World Health Organization, 2019). SNUD focuses on the interactive, social, communicative online activity, instead of highlighting one specific platform or the specific device while using a SNS (Wegmann et al., 2018).

The I-PACE (Interaction of Person-Affect-Cognition-Execution) model by Brand et al. (2016, 2019), is a theoretical framework investigating addictive behavior; it describes how predisposing variables interact with further cognitive and affective mechanisms, which could result in a loss of control when using a specific Internet application such as a SNS. The definition of predisposing variables to develop SNUD described in the I-PACE model includes psychopathological symptoms such as depression, anxiety, and interpersonal sensitivity to be a risk factor for the development and maintenance of an addictive behavior. In line with theoretical considerations, recent reviews have reported associations between SNUD and psychological factors. For instance, Kuss et al. (2014) reviewed epidemiological studies of SNUD and found that factors associated with SNUD appear to be complex. The factors included sociodemographic factors, (e.g., gender, family income), Internet usage factors (e.g., frequency and length of internet use), psychosocial factors (e.g., stress, emotional stability, and personality), and comorbid symptoms (e.g., alcohol use, depression, and anxiety; see Mythily et al., 2008; Liu et al., 2011). Furthermore, several studies have reported findings of SNUD being associated with negative consequences in peoples' lives such as poor sleep quality (e.g., Wolniczak et al., 2013; Xanidis and Brignell, 2016). Associations between psychopathological symptoms and SNUD have also been reported. Several studies have reported associations between SNUD and depression (e.g., Andreassen et al., 2016; Donnelly and Kuss, 2016; Sun et al., 2016; Wegmann and Brand, 2016; Shensa et al., 2017; Kircaburun et al., 2018). Furthermore, several studies have reported associations between SNUD and anxiety as well as interpersonal sensitivity (Wegmann and Brand, 2016; Lian et al., 2017; Oberst et al., 2017; Pontes, 2017; Van Rooij et al., 2017; Atroszko et al., 2018). These are just a few examples of studies on the topic of psychopathology and associations with SNUD symptoms. Altogether, these research findings show that SNUD may have implications for health and well-being (Andreassen and Pallesen, 2014; Zhou et al., 2017). Beyond that, most of the studies and reviews reporting associations between SNUD and psychopathological symptoms have been undertaken or reported on Western samples. This is a view too narrow-minded when considering the rise of SNSs in China, therefore we believe that a review of non-western SNS use and associations with psychopathological symptoms with a focus on China is much needed to gain a better understanding on this issue.

### Review Aims/Rationale

In sum, with the growing popularity of Chinese SNSs, a review of Chinese SNS use is much needed. We focus on Chinese SNSs in the present work, because with its hundreds of millions of users and mighty social media platforms such as Tencent's WeChat® it represents without doubt one of the most important digital forces in a connected world. In this part of the world, unintended side effects of digitization such as the development of addictive behaviors toward diverse online content need to be investigated (Montag and Diefenbach, 2018; Scholz et al., 2018). Furthermore, prevalence rates outline that SNUD seems to be a serious problem in Eastern cultures, especially when comparing those prevalence rates to Western cultures (e.g., Khumsri et al., 2015; Guedes et al., 2016; Stodt et al., 2018; Yang et al., 2019). We believe that a focus on Chinese SNSs is timely and relevant because much of what has been presented in the literature represents the Western view on the topic (including a strong focus on Western SNSs such as Facebook® see Sindermann et al., 2020). Therefore, reviewing literature on SNUD and associations with anxiety and depression from an Eastern perspective helps to get a more balanced view on the topic. Furthermore, Chinese SNS use is rapidly growing and focusing on Chinese SNS use is important because platforms such as WeChat® are not fully comparable to its Western equivalents (such as Facebook® or WhatsApp® see Montag et al., 2018a). Therefore, the question arises of whether associations between SNUD and psychopathological variables are valid both in Western and Eastern parts of the world. In the current review, we set out to discover whether associations exist between the above-mentioned variables in Chinese SNSs. Moreover, we were interested in gaining insights into the strength of associations. In order to accomplish this objective, a systematic review of Chinese SNSs use was conducted.

## Methods

### Search Strategy

**Figure 1:**
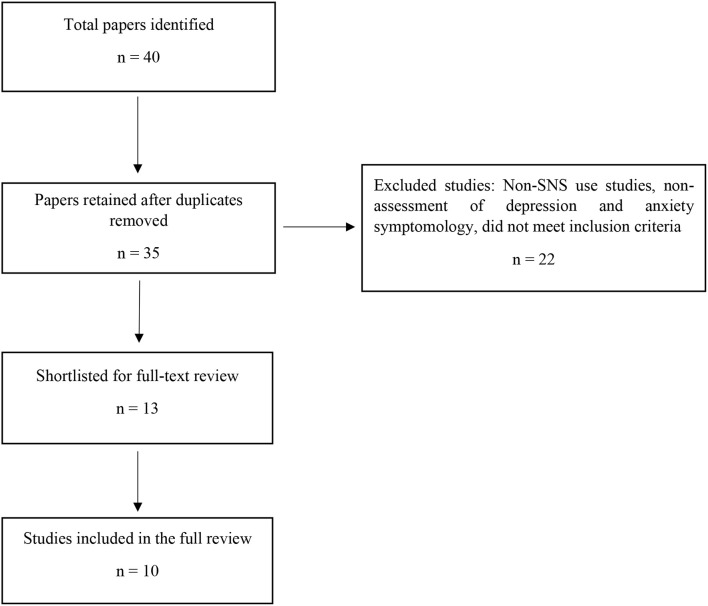
Flow diagram of the study selection process.

The preferred reporting items for systematic reviews and meta-analysis (PRISMA; Moher et al., 2010) were closely adhered to during the review process (see Figure 1). A systematic review of publications from January 2014 to June 2019 was conducted. The focus was on recent Chinese SNUD studies. Searches were completed on the following databases: PsycINFO, PsycARTICLES, Psychology and Behavioral Sciences Collection, MEDLINE, ProQuest, Web of Sciences, PubMed, Google Scholar, and the Chinese National Knowledge Infrastructure database (CNKI; this is a Chinese language database, the third author extensively searched the Chinese research literature). Terms to search for papers included “China” OR “Chinese,” and in combination with (using the AND Boolean operator) “social networking site” OR “social media” AND “patholog^*^ OR problem^*^ OR addict^*^ OR compuls^*^ OR dependen^*^ OR disorder^*^” AND “depression” OR “anxiety.” Finally, relevant journals were searched for recently added papers, including *Cyberpsychology, Behavior, and Social Networking, Journal of Behavioral Addictions*, and *Computers in Human Behavior*. Each study's title and abstract were screened for eligibility. Full texts of all potentially relevant studies were then retrieved and further examined for eligibility. Studies were systematically and independently reviewed by the authors and assessed regarding the study type, study population, methodology, measures used, and interpretation of the results.

### Inclusion/Exclusion Criteria

For studies to be included in the review, the following characteristics had to be met: (i) being published since 2014 onwards, (ii) having population-based studies, (iii) having specific criteria for SNUD (typically validated psychometric scales), (iv) containing empirical primary data reporting on the correlation between SNUD and the psychopathological variables of depression and anxiety, (v) examining any type of Chinese SNS use and, (vi) being published in English or Chinese/Mandarin. Studies were screened based on the titles and abstracts reporting on the topic of interest. Thereafter, studies were selected based on scientific relevance of the study and included following full-text assessment. They were evaluated if there was an observation of a full association or a finding of some type (i.e., an expected effect, null finding). Full association was considered when a correlation was found for SNUD and specific psychopathological symptoms following bivariate or multivariate analysis. The geographical distribution of studies was also mapped.

After deleting duplicate studies, a total of 35 papers were screened and identified via the systematic search strategy employed. As a result, 22 studies were excluded because they (i) dealt not with SNS use, (ii) did not assess depression or anxiety symptomology, and (iii) did not meet the aforementioned inclusion criteria. Three more studies were excluded after full text review of the studies (see Figure 1 for a flow diagram of the review process). In total, 10 papers were included in the present review. The characteristics of the studies included in the review (see Table 1) are discussed below. Some studies are referred to in more than one section due to assessing more than one psychopathological symptom. The results section also briefly reports the key findings of a further seven studies that met the inclusion criteria of assessing SNUD but did not assess psychopathological symptoms.

**Table 1:** Study details and results.

**Authors (year and place of study)**	**Sample size (age range)**	**Study variables**	**Scale used to assess SNUD**	**Results with SNUD**	**Effect sizes: bivariate results**	**Effect sizes: multivariate results**
Chen et al. (2019, China)	437 (16–30 years)	SNUD, anxiety	Social Networking Websites Addiction Scale (SNWAS; Turel and Serenko, 2012), Social Networking Sites Addiction Tendency Scale (SNSATS; Wilson et al., 2010)	Positive and significant association with anxiety	Anxiety *r* = 0.29	High anxiety was associated with high levels of SNUD (β simple slope = 0.32, *p* < 0.001)
Hong et al. (2014; Taiwan)	241 (18–22 years)	SNUD, depression, self-esteem, extraversion, neuroticism, sense of inferiority	Internet Addiction Test (Young, 1996)	Positive and significant association with depression	Depression *r* = 0.25	Depression significantly predicted SNUD; β = 0.211
Hou et al. (2019; China)	641 (17–25 years)	SNUD, depression, anxiety, perceived stress, resilience, social support	Facebook Intrusion Questionnaire (Elphinston and Noller, 2011)	Positive and significant association with depression and anxiety	Depression *r* = 0.22 Anxiety *r* = 0.22	Depression (β =0.14, 0.12, *p* < 0.05) and anxiety (β = 0.14, 0.12, *p* < 0.05) were positively associated with SNUD.
Li et al. (2017, China)	1,015 (7th−9th grade students: age range unreported)	SNUD, Internet use disorder, depression, insomnia,	Facebook Addiction Scale (Koc and Gulyagci, 2013)	Positive and significant association with depression	Depression *r* = AOR = 3.27, 95% CI: 2.33, 4.59)	Insomnia partially mediated 44.8% of the effect of SNUD on depression (Sobel *Z* = 3.919, *p* < 0.001)
Li et al. (2018a; 2018b, China)	5,365 (mean age = 13.9 years in the longitudinal sample: age range unreported)	SNUD, depression	Online Social Networking Addiction Scale (Li et al., 2016)	Positive and significant association with depression	Baseline SNUD was significantly associated with higher incidence of depression during the follow-up period (univariate OR: 1.65, 95% CI: 1.22–2.22).	As compared to adolescents without depression, the odds of developing SNUD were 3.45 times (95% CI: 2.51–4.75) higher among those who were persistently depressed, and 4.47 times (95% CI: 3.33–5.99) higher among those who were emerging depressed
Liu and Ma (2018a, China)	519 (Male mean age 19.42, female mean age 18.81: age range unreported)	SNUD, anxiety, SNS burnout, envy	Social Media Addiction Scale (Liu and Ma, 2018b)	Positive and significant association with anxiety	Anxiety *r* = 0.56	SNUD is a significant predictor of anxiety. Anxiety was a mediator between SNUD and burnout [mediation effect = 0.0795 (95% CI, (0.0546, 0.1075)].
Niu et al. (2018, China)	746 (12–18 years)	SNS intensity, depression, negative social comparison, self-esteem	Facebook Intensity Scale (Ellison et al., 2007)	Positive and significant association with depression	Depression *r* = 0.206	Indirect effect of negative social comparison in the relationships between SNS use and depression (Mediating effect = 0.050, *SE* = 0.009 Bootstrap 95% CI: 0.032/0.086)
Tian et al. (2018, China)	5,215 (10–23 years)	SNS intensity, depression, loneliness, life satisfaction, Internet gaming, online pornography	Facebook Intensity Scale (Ellison et al., 2007)	Negative and significant association with depression	Depression *r* = −0.08	3% of the variance of depression was explained by social networking site use (β = −0.06, *p* < 0.01)
Wang et al. (2018, China)	365 (14–18 years)	SNUD, depression, rumination, self-esteem	Facebook Intrusion Questionnaire (Elphinston and Noller, 2011)	Positive and significant association with depression	Depression *r* =0.18	SNUD positively predicted depression, β = 0.18, *p* < 0.001
Yam et al. (2019, Hong Kong)	307 (17–30 years)	SNUD, gaming disorder, depression, anxiety	Bergen Social Media Addiction Scale (Andreassen et al., 2012)	Positive and significant associations with depression and anxiety	Depression *r* = 0.18 Anxiety *r* = 0.19	n/a

*Effect sizes are factor results reported with SNUD unless otherwise stated; β, standardized regression coefficient; B, Unstandardized regression coefficient; AOR, Adjusted odds ratio; CI, Confidence interval*.

## Results

### Description of Included Studies and Geographical Distribution

Nine studies were cross-sectional survey studies, only the study by (Li et al., 2018a,b) used a prospective cohort study design. All ten of the studies targeted adolescents and/or emerging adult groups. All studies examined both genders and the sample sizes ranged from 241 to 5,365. Most studies (*n* = 8) were carried out in Mainland China, one study was carried out in Hong Kong (Yam et al., 2019), and one study was carried out in Taiwan (Hong et al., 2014). Given that China, Taiwan, and Hong Kong share the same cultural background, we deemed it important to include studies from these areas of the world in the present review. Furthermore, the participants in these studies were Chinese SNS users. Table 1 summarizes further information about the included studies and provides insights into the effect sizes observed in each study.

### Methods of Assessing Social Networks Use Disorder

Various measures were used to assess SNUD with the authors of studies adapting measures into Chinese/Mandarin and to assess Chinese SNSs (i.e., western SNS name, such as Facebook®, was exchanged for a Chinese SNS name such as WeChat®). Hou et al. (2019) and Wang et al. (2018) utilized adapted versions of the Facebook Intrusion Questionnaire (Elphinston and Noller, 2011; Li et al., 2018a,b) used the Online Social Networking Addiction Scale (Li et al., 2016), Liu and Ma (2018a) used the Social Media Addiction Scale (Liu and Ma, 2018b), Chen et al. (2019) used the Social Networking Websites Addiction Scale (SNWAS; Turel and Serenko, 2012) and the Social Networking Sites Addiction Tendency Scale (SNSATS; Wilson et al., 2010). (Yam et al., 2019) used the Bergen Social Media Addiction Scale (Andreassen et al., 2012), Li et al. (2017) used an adapted version of the Facebook Addiction Scale (Koc and Gulyagci, 2013), and Hong et al. (2014) used the Internet Addiction Test (Young, 1996). Two studies (Niu et al., 2018; Tian et al., 2018) used an adapted version of the Facebook Intensity Scale (Ellison et al., 2007) to assess SNS use intensity. Although not a direct measure of SNUD, research has reported that SNS intensity is related to addiction, therefore it appears to be a valid measure of SNUD (Müller et al., 2016). The measures for assessing SNUD varied, some studies had different measurement criteria. Table 1 provides details of measurement instruments used by the studies to assess SNUD.

### Social Networks Use Disorder and Depression Symptoms

Eight studies examined the associations between SNUD and depression (i.e., Hong et al., 2014; Li et al., 2017, 2018a,b; Niu et al., 2018; Tian et al., 2018; Wang et al., 2018; Hou et al., 2019; Yam et al., 2019). A significant and positive association between SNUD and depression was reported in seven studies (i.e., Hong et al., 2014; Li et al., 2017, 2018a,b; Niu et al., 2018; Wang et al., 2018; Hou et al., 2019; Yam et al., 2019). Tian et al. (2018) reported a significant negative association between SNUD and depression. Bivariate correlations were typically in the range from −0.08 to 0.259. Two studies (Li et al., 2017, 2018a,b) reported odds ratios, which were in the range of 1.65–3.27. Multivariate associations showed betas ranging from −0.06 (Tian et al., 2018) to 0.211 (Hong et al., 2014). Li et al. (2017) reported that insomnia partially mediated 44.8% of the effect of SNUD on depression (Sobel *Z* = 3.919, *p* < 0.001). One study reported that the odds of developing SNUD was 3.45 times (95% CI: 2.51–4.75) higher among those who were persistently depressed, and 4.47 times (95% CI: 3.33–5.99) higher among those who were emerging depressed (Li et al., 2018a,b). Several scales were used to assess depression in the studies. Most of the studies (Li et al., 2017, 2018a,b; Niu et al., 2018; Tian et al., 2018; Wang et al., 2018) made use of the Chinese version of the Center for Epidemiological Studies Depression Scale (CES-D; Chen et al., 2009). Hong et al. (2014) used the depressive character sub-scale of Lai's Personality Scale (Lai and Lai, 2003). Yam et al. (2019) used the Hospital Anxiety and Depression Scale (HADS; Chan et al., 2010).

### Social Networks Use Disorder and Anxiety Symptoms

Four studies examined the associations between SNUD and anxiety (i.e., Liu and Ma, 2018a; Chen et al., 2019; Hou et al., 2019; Yam et al., 2019). A significant, positive association between SNUD and anxiety was reported in four studies (i.e., Liu and Ma, 2018a; Chen et al., 2019; Hou et al., 2019; Yam et al., 2019). Bivariate correlations were typically in the range of 0.19–0.56. Three of the studies reported inferential statistical results (i.e., Chen et al., 2019—high anxiety was associated with high levels of SNUD (β simple slope =.32, *p* < 0.001); (Liu and Ma, 2018a)—SNUD was a significant predictor of anxiety, anxiety was a mediator between SNUD and burnout (mediation effect = 0.0795 (95% CI, [0.0546, 0.1075]); (Hou et al., 2019)—anxiety was positively associated with SNUD (β = 0.14, 0.12, *p* < 0.05). Several scales were used to measure anxiety in the studies. Chen et al. (2019) used the Chinese version of the Social Phobia Scale (Ye et al., 2007). Hou et al. (2019) used the State-Trait Anxiety Inventory (Spielberger et al., 1970). Liu and Ma (2018a) used the Social Anxiety Scale for Social Media Users (SAS-SMU; Alkis et al., 2017). Yam et al. (2019) used the Hospital Anxiety and Depression Scale (HADS; Chan et al., 2010).

### Social Networks Use Disorder and Further Predisposing Variables in Chinese Samples (Not Meeting the Inclusion Criteria)

In line with the I-PACE model (Brand et al., 2016, 2019), further predisposing variables have been identified as risk factors that are associated with SNUD. Thus, during the initial literature search, seven studies were found that examined SNUD among Chinese SNS users but did not meet the inclusion criteria (other predisposing variables were found to be associated with SNUD). We briefly describe these studies here. Lian et al. (2018) examined associations between SNUD, irrational procrastination, SNS fatigue, and effortful control. Results indicated that SNUD, irrational procrastination, and SNS fatigue were positively correlated with each other, and negatively correlated with effortful control. Further analysis revealed that SNUD had a direct effect on irrational procrastination. Montag et al. (2015) reported correlations between SNUD and unspecified Internet-use disorder amongst Chinese and Taiwanese samples. Hou et al. (2018) assessed how personality traits and psychological factors relate to excessive use of WeChat® and Weibo®. The results showed that addictive use of Weibo® and WeChat® correlated positively with neuroticism, loneliness, and external locus of control and negatively with agreeableness, social support, and social interaction. Li et al. (2018a,b) examined influences of stressful life events and problematic use of WeChat® on life satisfaction. The results showed that stressful life events were positively associated with addictive use of WeChat®. Zhou and Wang (2017) explored the relationships between addictive use of WeChat® and self-control. The results outlined a significant negative correlation between addictive use of WeChat® and self-control. Liu and Ma (2018b) reported that SNUD symptoms were positively correlated with the pathological use of the smartphone, pathological Internet use, and narcissism, but negatively correlated with self-esteem. Wang et al. (2015) reported that SNUD was significantly associated with neuroticism and extraversion.

## Discussion

The present systematic review investigated SNUD and its associations with depression and anxiety symptoms in ten studies examining Chinese SNS use that met the inclusion criteria. A review of SNUD in Eastern cultures was much needed as there is a lack of research focusing on this topic as well as on possible convergent and divergent mechanisms between Eastern and Western cultures. The current review emphasizes that SNUD co-occurs with psychopathological symptoms. Most associations were found between SNUD and depression symptoms (Yu et al., 2018), but effect sizes were higher between SNUD and anxiety. Considering SNUD and comparisons between Western and Eastern cultures, the results are comparable to the review by Hussain and Griffiths (2018) who found that SNUD was associated with depression and anxiety symptomology in several European studies. More specifically, several Western studies (Andreassen et al., 2016; Pontes, 2017; Shensa et al., 2017; Van Rooij et al., 2017; Kircaburun et al., 2018; Worsley et al., 2018) have reported associations between SNUD and depression symptoms with small to moderate effect sizes. These effect sizes are similar to the Chinese SNUD studies reported in this review. Several Western studies (Andreassen et al., 2016; Pontes, 2017; Van Rooij et al., 2017; Atroszko et al., 2018; Worsley et al., 2018) have reported associations between SNUD and anxiety symptoms with small effect sizes. These effect sizes are similar to the Chinese SNUD studies reported in this review (although these were higher compared to associations with depression). A recent meta-analysis by Marino et al. (2018) examining the associations between SNUD and psychological distress reported a medium bivariate effect size. These findings are similar to the effect sizes reported in the present review. Other meta-analyses (Huang, 2012; Song et al., 2014) have reported small effect sizes, which differs to the effect sizes reported for Chinese SNS use, which were higher. In sum, the present review evidenced statistically significant associations between SNUD and depression and anxiety symptoms. However, the effect sizes reported were higher than a recent meta-analysis examining Internet use and well-being (Çikrikci, 2016) and consistent with a meta-analysis investigating problematic Internet use and social anxiety (Prizant-Passal et al., 2016). Nevertheless, please note that (a) we did not conduct a meta-analysis and (b) well-being in terms of life satisfaction or positive emotionality was not the focus of the present review work, and there are few related studies (Zhou et al., 2017).

The results outline that overall the associations between SNUD and psychopathological symptoms seem to be comparable in Western as well as Eastern cultures. Nevertheless, research on SNUD and the addictive use of the Internet in general should go a step further. Investigating the relationship between psychopathology and SNUD is important for gaining an initial understanding of this potential disorder. However, bivariate correlations do not allow conclusions about underlying mechanisms or the process of the development and maintenance of addictive Internet use or SNUD. Moreover, it gives no answers to what negative consequence appeared first. Therefore, the question remains if psychopathological symptoms present a prerequisite to develop SNUD and/or if they represent a consequence of an addictive behavior. Consistent with the theoretical considerations of the I-PACE model (Brand et al., 2016, 2019), the interaction of predisposing variables such as depression, anxiety, and interpersonal sensitivity with further affective and cognitive mechanisms should be investigated in Eastern cultures as well. For example, the empirical study by Wegmann and Brand (2016) already outlined that aside from bivariate correlations the effect of psychopathological symptoms on tendencies toward SNUD is mediated by use expectancies, and similar studies have yielded the same conclusions (Niu et al., 2016a). The expectancies to escape from negative emotions and to experience pleasure by using SNSs seem to represent a reinforcement mechanism, which enhances the risk of individuals developing problematic use of SNSs or other communication applications. The interactive effect of predisposing variables and cognitive and affective mechanisms is discussed in several studies as well as in theoretical models (e.g., Niu et al., 2016b; Wegmann and Brand, 2016; Brand et al., 2019). Understanding these reinforcement mechanisms are relevant for the definition of convergent and divergent mechanisms in different specific forms of Internet-use disorders as well as in different cultures (Yao et al., 2014). This could also affect preventive mechanisms and treatment programs in Western and Eastern cultures.

Stodt et al. (2018) showed that the relevance of Internet-literacy capabilities as a preventive and protective role for the development and maintenance of addictive use of the Internet in general seem to differ in Germany and China. Additionally, Lachmann et al. (2018) outline comparable associations between symptom severity of addictive use of the Internet and life satisfaction and empathy. However, taking a closer look, the results also emphasize that there are differences in Chinese and German students. It could be speculated that the effect of protective factors such as high empathy or life satisfaction differ in both cultures, although works such as by Lachmann et al. (2018) or older work by Melchers et al. (2015) rather hint at the comparability of mechanisms. In general, the present review results are in line with the study by Yang et al. (2019) comparing British and Chinese students; our findings emphasize differences regarding prevalence rates between cultures.

Further research is needed which especially examines the reinforcement mechanisms of the development and maintenance of SNUD as well as other types of Internet-use disorders in both cultures. SNUD is likely to influence the health and well-being of SNS users; it may also be the case that people with high levels of depression and anxiety may end up displaying maladaptive technology behaviors. However, this is currently a speculative assumption; the reviewed studies cannot answer these assumptions due to the cross-sectional nature of the studies. Future longitudinal studies will help to establish causal relationships. Distinguishing between Eastern and Western cultures and SNUD for future research questions is one way forward as the online applications are different in these cultures. The specific uses of SNSs may be different between cultures. Furthermore, Asian users have been shown to display more SNUD symptoms than Western users (Kuss et al., 2014; Stodt et al., 2018; Yang et al., 2019). This shows that further research examining SNUD in Asian countries is warranted. In addition, future research should consider other risk factors that may be associated with SNUD, such as impulsivity, and neuroticism (see recent works by Elhai et al., 2019; Peterka-Bonetta et al., 2019; Sha et al., 2019). Examining specific uses of SNSs (e.g., social interaction, posting photo's, viewing the profiles of other users) and its impact on psychological well-being is an important area for future research (see Rothen et al., 2018; Twenge et al., 2018).

### Limitations and Future Research

Most of the reviewed studies used self-report methods and were cross-sectional, which makes it difficult to identify causal associations. Furthermore, specific activities engaged in by SNS users were not recorded making it difficult to ascertain the causes of SNUD, depression, and anxiety. The reviewed studies consisted of adolescent samples who tend to be the main users of this technological medium. This said, older people use SNSs and therefore studies examining SNS use among older users is warranted as this is an under-studied age-group. The current review did not investigate other relevant variables such as obsessive-compulsive disorders and loneliness; future research could investigate these variables. It is important to note that different measures were used to assess SNUD in the reviewed studies and therefore researchers had different criteria for assessing SNUD. Currently, no diagnostic standard exists to assess and diagnose SNUD, even if it is seen to be mandatory to increase consistency of measurement in this field of research. However, there are unanswered questions regarding causality and underlying factors regarding the occurrence of symptoms (of both SNUD and psychiatric disorders). Prospective studies will help to answer these questions (Starcevic and Khazaal, 2017). Information on SNS use among individuals diagnosed with depression or anxiety disorders are limited (Prizant-Passal et al., 2016), future studies that utilize clinical samples to examine the SNUD-psychopathology relationship is much needed. Beyond that, it will be of large importance to also study the design of social media/messenger platforms both in the Western and Asian world in order to understand which in-built elements, such as Likes or retweets, actually foster addictive behaviors (Montag et al., 2019b). Finally, the low number of studies in the review means that additional reviews are warranted when significantly more studies have been published. Nevertheless, for the moment we believe our work to be comprehensive and it addressed an important gap in the research literature.

## Conclusions

The present review revealed associations between SNUD and psychopathological symptoms among Chinese SNS users. A review of SNUD in Eastern cultures was warranted due to a lack of knowledge about SNS use in China. The findings were insightful and have the potential to inform prevention and interventions on SNUD in Eastern cultures and will be of benefit to researchers studying the impact of SNUD.

## Author Contributions

ZH, EW, and CM designed the present study. ZH wrote the first draft of the present work and carried out the systematic review. HY reviewed the scientific literature written in Mandarin. All authors worked over the first draft and approved the final version.

### Conflict of Interest

The authors declare that the research was conducted in the absence of any commercial or financial relationships that could be construed as a potential conflict of interest.
